# A Modern‐Day Captain Charles Martell: Catastrophic Skeletal Manifestations of Primary Hyperparathyroidism

**DOI:** 10.1155/crie/1352595

**Published:** 2026-05-14

**Authors:** Rafyat Ara, Ahmad Alam, Dileep Raja Kuraku, Mohd Salman, Vijayakumar Karthik

**Affiliations:** ^1^ Department of Medicine, Jawahar Lal Nehru Medical College, Aligarh Muslim University, Aligarh, 202002, Uttar Pradesh, India, amu.ac.in; ^2^ Rajiv Gandhi Centre for Diabetes and Endocrinology, Jawahar Lal Nehru Medical College, Aligarh Muslim University, Aligarh, 202002, Uttar Pradesh, India, amu.ac.in; ^3^ National Pirogov Memorial University, Pyrohova Street 56, Vinnytsia, 21018, Vinnytsia Oblast, Ukraine; ^4^ Department of Endocrinology, NIMS Medicity, Thiruvananthapuram, 695004, Kerala, India

**Keywords:** hungry bone syndrome, hypercalcemia, osteitis fibrosa cystica, parathyroid adenoma, pathologic fractures, primary hyperparathyroidism

## Abstract

Primary hyperparathyroidism (PHPT) is characterised by autonomous overproduction of parathyroid hormone (PTH), resulting in hypercalcemia and multisystem complications. Although routine calcium screening has reduced advanced skeletal disease in many regions, severe manifestations such as osteitis fibrosa cystica (OFC) still occur in resource‐limited settings. We report a case of a woman in her 30s who presented with profound disability after 4–5 years of progressive bone pain, fatigue, proximal myopathy and eventual loss of independent ambulation, requiring her to drag herself on the ground. Imaging revealed generalised osteopenia, subperiosteal bone resorption, a ‘salt and pepper’ appearance of the skull, codfish vertebrae, anterior bowing of long bones and multiple lytic lesions consistent with advanced OFC. Laboratory investigations revealed marked hypercalcemia (14.3 mg/dL), hypophosphataemia (1.49 mg/dL), elevated alkaline phosphatase (781 U/L) and elevated intact PTH (iPTH) (1110 pg/mL), confirming PTH‐dependent hypercalcemia. A 4D‐CT scan localised a left inferior parathyroid adenoma (LIPA), and a renal ultrasound showed bilateral nephrocalcinosis. Following preoperative stabilisation with saline diuresis and intravenous zoledronic acid, she underwent minimally invasive parathyroidectomy (MIP) with an immediate postoperative fall in PTH, indicating biochemical cure. However, she developed hungry bone syndrome (HBS), requiring intensive intravenous and oral calcium supplementation along with active vitamin D therapy. This case highlights the catastrophic skeletal consequences of delayed PHPT diagnosis. It underscores the importance of early recognition of metabolic bone disease to prevent irreversible disability and systemic complications, particularly in low‐resource settings.

## 1. Introduction

Primary hyperparathyroidism (PHPT) is a common endocrine disorder characterised by the autonomous overproduction of parathyroid hormone (PTH), leading to hypercalcemia and a broad spectrum of skeletal, renal, gastrointestinal and neuropsychiatric complications. Before the era of routine biochemical screening, patients frequently presented with advanced manifestations such as osteitis fibrosa cystica (OFC), pathological fractures and nephrolithiasis [1].

One of the earliest and most illustrative cases of PHPT was reported in the 1920s and involved Captain Charles Martell, a physically robust merchant marine officer who developed generalised bone pain, multiple low‐trauma fractures, nephrolithiasis and profound skeletal deformities. Despite being evaluated by numerous physicians, a diagnosis of ‘hyperactivity of the parathyroid bodies’ was delayed by 7 years. He underwent multiple unsuccessful neck explorations before a retrosternal parathyroid adenoma was finally identified and excised. Sadly, he ultimately succumbed to complications of obstructive uropathy. His case played a pivotal role in advancing the early understanding of PHPT and its systemic consequences [2].

With the advent of routine calcium screening, such florid skeletal manifestations have become exceedingly rare in contemporary clinical practice. However, in resource‐limited settings, delayed diagnosis may still lead to classical presentations of advanced PHPT [3]. We report the case of a woman in her 30s with longstanding, untreated PHPT, who developed severe skeletal deformities, pathological fractures and complete loss of independent mobility. This case provides a rare glimpse into the severe end of the PHPT spectrum and underscores the critical importance of early diagnosis, particularly in low‐resource settings.

## 2. Case Presentation

A female in her 30s presented to the endocrinology outpatient department in a profoundly disabled state. She was unable to stand or walk and had to move by dragging herself on the ground, using her hands for support while her hips remained in contact with the floor. Her lower limbs were aligned horizontally with the ground, and she exhibited a noticeable short stature, short neck and shrunken torso. Examination revealed anterior bowing of both legs and clinical evidence of proximal myopathy. Her symptoms began approximately 4–5 years prior to presentation, initially with an insidious onset of generalised body aches and fatigue. Over the next 1–2 years, she developed progressive proximal muscle weakness, with increasing difficulty in climbing stairs and rising from a seated position. During this period, she sought medical attention multiple times and was treated empirically with calcium, vitamin D and multivitamin supplements without undergoing biochemical evaluation. Over the subsequent 2 years, her symptoms worsened, and she gradually became dependent on support for ambulation. Approximately 9 months prior to presentation, she sustained a pathological fracture of the left proximal femur following trivial trauma, which was managed surgically with intramedullary fixation. However, no metabolic evaluation was performed at that time to identify the underlying cause. Her condition continued to deteriorate, eventually leading to complete loss of independent mobility, necessitating crawling for movement and full dependence for activities of daily living.

**Figure 1:**
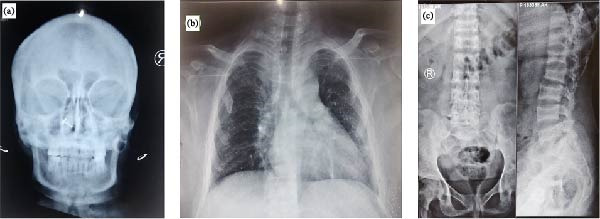
(a) Skull radiograph demonstrating calvarial thickening with a granular ‘salt‐and‐pepper’ appearance and subcortical resorption around the mandibular tooth sockets with loss of lamina dura. (b) Chest radiograph demonstrating diffuse bony rarefaction with an accentuated trabecular pattern and subchondral resorption at the costochondral junctions. (c) X‐ray of the lumbosacral spine (AP and lateral views) showing generalised osteopenia with biconcave vertebral bodies consistent with codfish vertebrae and reduction in vertebral body heights.

**Figure 2:**
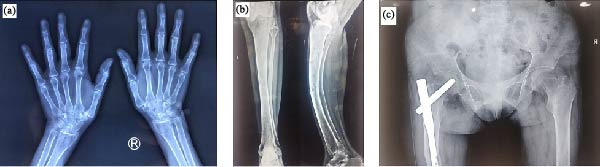
(a) Bilateral hand radiographs demonstrating subperiosteal resorption along the middle phalanges. (b) X‐ray of the left tibia (AP and lateral views) showing a well‐defined lytic lesion in the mid‐shaft with a narrow zone of transition and surrounding sclerosis, suggestive of a healed Brown’s tumour. (c) X‐ray pelvis showing a left femoral implant in situ with pubic symphysis resorption and generalised osteopenia characterised by coarse, prominent trabeculae, most marked in the bilateral iliac bones.

**Figure 3:**
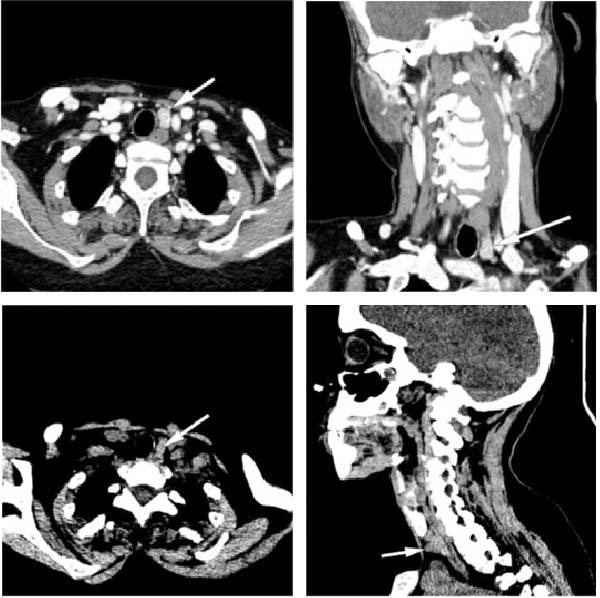
Contrast‐enhanced CT of the neck (axial, coronal and sagittal reconstructions) demonstrates a well‐defined, avidly enhancing lesion measuring approximately 1.3 cm × 2.0 cm, located posterior to the left thyroid lobe, consistent with a left‐sided parathyroid adenoma.

The skeletal survey revealed generalised osteopenia with coarse trabecular patterns and multiple lytic lesions. Skull radiographs showed calvarial thickening with a granular, mottled appearance, loss of distinction between the inner and outer tables and subcortical resorption around the mandibular tooth sockets (Figure 1a). The chest X‐ray showed a shortened thoracic cage with subchondral resorption at the costochondral junctions (Figure 1b), while spine films revealed codfish vertebrae (Figure 1c). The X‐rays of bilateral wrists demonstrated subperiosteal bone resorption, collectively representing classical features of OFC (Figure 2a). X‐rays of both tibiae and fibulae (anteroposterior and lateral views) revealed anterior bowing, mild cortical thinning and irregular endosteal margins (Figure 2b). Pelvic imaging demonstrated a pathological fracture of the left proximal femur with internal fixation (Figure 2c). Given the patient’s skeletal deformities, proximal muscle wasting and history of pathological fracture, metabolic bone disease was strongly suspected. Laboratory evaluation revealed severe hypercalcemia with a serum calcium level of 14.3 mg/dL (reference: 8.6–10.2 mg/dL), hypophosphataemia with phosphate at 1.49 mg/dL (reference: 2.5–4.5 mg/dL), and elevated alkaline phosphatase at 781 U/L (reference: 30–130 U/L). Serum creatinine was 1.2 mg/dL (0.6–1.2 mg/dL), with an eGFR of 61 mL/min/1.73 m^2^ (CKD‐EPI; reference >90 mL/min/1.73 m^2^), and serum 25‐hydroxyvitamin D was 21 ng/mL (30–100 ng/mL). Serum intact PTH (iPTH) was markedly raised to 1110 pg/mL (reference: 15–65 pg/mL), indicating PTH‐dependent hypercalcemia consistent with a diagnosis of PHPT. A 4D‐CT scan localised a left inferior parathyroid adenoma (LIPA) (1.3 cm × 2.0 cm) (Figure 3), and renal ultrasound revealed bilateral nephrocalcinosis, with the largest calculus measuring 7.6 mm. Baseline dual‐energy X‐ray absorptiometry (DEXA) showed BMD values of 0.574 g/cm^2^ (*Z*‐score −4.0) at the lumbar spine (L1–L4), 0.532 g/cm^2^ (*Z*‐score −3.6) at the left femoral neck and 0.395 g/cm^2^ (*Z*‐score −4.5) at the forearm, consistent with bone density below the expected range for age. Given the relatively young age at presentation, multiple endocrine neoplasia type 1 (MEN1) was considered. However, there was no history of menstrual irregularity or galactorrhoea, and serum prolactin was 12 ng/mL (reference range 5–25 ng/mL). Genetic testing for a familial or syndromic aetiology was not performed due to financial constraints.

### 2.1. Management and Follow‐Up

**Figure 4:**
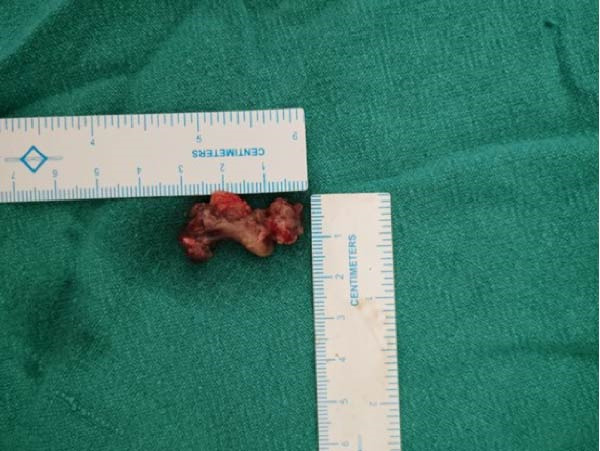
Gross specimen of the excised left inferior parathyroid adenoma obtained following unilateral neck exploration, shown alongside a centimetre scale for size reference.

She was preoperatively managed with intravenous normal saline (3 L/day for 3 days) to achieve adequate hydration and promote calciuresis, along with a single dose of 4 mg of intravenous zoledronic acid. This resulted in a reduction in serum calcium from 14.3 to 9.2 mg/dL prior to surgery. After stabilisation, the patient underwent minimally invasive parathyroidectomy (MIP) for excision of the LIPA. Intraoperatively, a well‐circumscribed, enlarged left inferior parathyroid gland was identified and removed. The lesion appeared reddish‐brown and soft, measuring approximately 2.5 cm × 1.5 cm (Figure 4). The immediate postoperative iPTH level dropped to 45 pg/mL, confirming biochemical cure and successful resection. On postoperative day 3, she developed hungry bone syndrome (HBS), with serum calcium falling to 6.7 mg/dL in association with hypophosphataemia, with serum phosphate measured at 1.3 mg/dL. She was treated with intravenous calcium gluconate, delivering approximately 450 mg of elemental calcium per day for 10 days, along with oral elemental calcium (3 g/day as calcium carbonate), cholecalciferol (60,000 units weekly) and calcitriol (1.0 µg/day). At discharge, her serum calcium was 8.6 mg/dL, her serum creatinine was 0.8 mg/dL and her eGFR was 98 mL/min/1.73 m^2^, and she was asymptomatic. She was discharged after a 3‐week hospital stay. Histopathological examination revealed a well‐circumscribed parathyroid lesion composed predominantly of chief cells arranged in sheets and nests. There was no evidence of capsular or vascular invasion, mitotic activity or necrosis. These findings were consistent with a parathyroid adenoma.

At 1‐year follow‐up, the patient demonstrated significant clinical improvement and was able to ambulate with a walking stick. Repeat DEXA showed improvement in bone mineral density across all sites (Table 1). Biochemically, serum calcium was 9.4 mg/dL, phosphate 2.9 mg/dL and alkaline phosphatase 196 U/L. She was maintained on oral elemental calcium 1.0 g/day and vitamin D supplementation at 60,000 units monthly.

**Table 1:** Changes in bone mineral density at 1‐year follow‐up.

Site	Baseline BMD (g/cm^2^)	1‐Year BMD (g/cm^2^)	Absolute change	% Change
Lumbar spine	0.574	0.650	+0.076	+13
Femoral neck	0.532	0.550	+0.018	+3
Forearm	0.395	0.420	+0.025	+6

## 3. Discussion

This case highlights the catastrophic skeletal manifestations of longstanding, untreated PHPT in a young woman in her 30s. The striking clinical and radiological features illustrate the classic presentation of OFC that has become exceedingly rare in contemporary medical practice.

OFC represents the most severe skeletal complication of untreated hyperparathyroidism, characterised by excessive osteoclastic bone resorption driven by persistently elevated PTH [4]. With the advent of routine biochemical screening in the latter half of the 20th century, severe skeletal disease has become increasingly uncommon, particularly in developed nations, where most patients are diagnosed at an asymptomatic stage [5]. Although such advanced manifestations are now rarely encountered in high‐income countries, they are still observed in developing and resource‐limited regions. Data from the Indian PHPT Registry demonstrate that up to 95% of patients present with symptomatic disease, with over half exhibiting skeletal involvement [6]. Limited access to routine serum calcium testing, low clinical suspicion and fragmented healthcare pathways often contribute to delayed diagnosis. Consequently, patients may still present with advanced disease, including OFC, pathological fractures and profound functional disability, as seen in our case.

The radiographic findings in this patient demonstrate the classic features of advanced OFC. The generalised osteopenia with accentuated trabecular markings results from increased osteoclastic bone resorption. The mottled ‘salt and pepper’ appearance of the skull, with loss of distinction between inner and outer tables, occurs due to demineralisation and replacement of mineralised bone with fibrous tissue. Brown tumours, which are non‐neoplastic but locally aggressive lesions composed of fibrous tissue, osteoid and multinucleated giant cells, appeared as well‐defined lytic areas, particularly in the tibiae [7]. The pathological fracture of the proximal femur reflects the structural compromise associated with severe skeletal demineralisation, while anterior bowing of the lower limbs likely results from chronic weight‐bearing on mechanically weakened and softened bone.

The extreme clinical presentation of crawling mobility, short stature and proximal myopathy illustrates the devastating consequences of prolonged hyperparathyroidism. Proximal muscle weakness arises from PTH‐induced mitochondrial dysfunction (via impaired fatty acid oxidation) and proteolytic muscle atrophy, compounded by chronic hypophosphataemia, depleting intracellular ATP reserves [7]. The patient’s short stature and shrunken torso likely resulted from vertebral compression fractures and rib cage deformities.

Renal involvement is a common manifestation of PHPT. While the prevalence of nephrolithiasis in Western populations has declined significantly from nearly 80% to 7%–20% over time, primarily due to early detection and intervention, studies from India continue to report a wide prevalence range (10%–70%) for nephrolithiasis or nephrocalcinosis in PHPT patients [8].

HBS developed predictably after parathyroidectomy. It results from rapid skeletal remineralisation following the abrupt normalisation of PTH levels, leading to profound hypocalcaemia. Risk factors include markedly elevated preoperative alkaline phosphatase levels, severe radiographic bone disease and significantly raised PTH levels, all of which were present in our patient. Management involved both intravenous and oral calcium supplementation, along with active vitamin D (calcitriol) to enhance intestinal calcium absorption [9].

Preoperative optimisation with saline diuresis and zoledronic acid helped stabilise hypercalcemia before surgery, an important step in perioperative management. While some studies suggest that bisphosphonates may mitigate the severity of postoperative HBS by reducing bone turnover, their routine use remains controversial due to inconsistent outcomes in the literature [9, 10].

Long‐term follow‐up shows gradual radiographic improvement. Brown tumours and lytic lesions typically remineralise within 3–6 months postoperatively, although residual deformities (e.g., tibial bowing) may persist [11, 12]. Muscle function generally improves with time and rehabilitation, and most patients regain ambulatory capacity within 6–12 months, although chronic atrophy may necessitate prolonged physiotherapy [13].

Notably, this case highlights multiple missed opportunities for early diagnosis, particularly during the initial phase of progressive musculoskeletal symptoms and at the time of pathological fracture, where even a basic serum calcium measurement could have led to early identification of hypercalcaemia. These observations emphasise the importance of early recognition of PHPT in patients presenting with unexplained bone pain, proximal myopathy or pathological fractures. From a clinical perspective, initial screening with serum calcium should be performed in such scenarios; if abnormal, timely referral and comprehensive biochemical evaluation, including serum phosphate, alkaline phosphatase, vitamin D and PTH, are essential to establish the diagnosis and prevent progression to advanced skeletal disease, especially in resource‐limited settings.

The patient’s vivid clinical and radiological features provide a rare and valuable educational opportunity. Most clinicians today may never encounter such a fully developed manifestation of OFC, rendering this case particularly impactful for teaching purposes.

## 4. Patient’s Perspective

Before coming to the hospital, I had been suffering from body pain and weakness for many years. Initially, my symptoms were mild, but over time they became so severe that I was unable to stand or walk. I gradually became dependent on others for even my daily activities and had to move by dragging myself on the ground. This was physically exhausting and emotionally distressing for me. After proper evaluation, I was diagnosed with a condition affecting my bones and calcium levels, and this diagnosis brought relief and hope. Following surgery and treatment, I experienced gradual improvement in strength and overall health. With continued support, I am now able to walk with assistance, an achievement that has helped me regain my independence and confidence.

## Author Contributions

Rafyat Ara was involved in clinical management of the patient, contributed to data collection and follow‐up and assisted in literature review and manuscript editing. Ahmad Alam was involved in clinical management, contributed to the conception and design of the case report and draughted the manuscript. Mohd Salman involved in clinical managementand contributed to data collection and follow‐up. Vijayakumar Karthik contributed to the conception and design of the case report and assisted in literature review and manuscript editing. Dileep Raja Kuraku assisted in literature review and manuscript editing. Mohd Salman is the guarantor of the work and takes full responsibility for the integrity of the data.

## Acknowledgments

The authors have nothing to report.

## Funding

No funding was received for this manuscript.

## Disclosure

All authors have read and approved the final manuscript.

## Consent

We confirm that written informed consent was obtained from the patient for publication of the case details and accompanying images.

## Conflicts of Interest

The authors declare no conflicts of interest.

## Data Availability

The data that support the findings of this study are available upon request from the corresponding author. The data are not publicly available due to privacy or ethical restrictions.
